# A Pathogenic ROCK-Signaling Network Involving a Lysine Deletion in Myh11 Renders Carriers Susceptible to Aortic Dissection

**DOI:** 10.3390/ijms27073195

**Published:** 2026-03-31

**Authors:** Hironori Okuhata, Shota Tomida, Tamaki Ishima, Ryozo Nagai, Kenichi Aizawa

**Affiliations:** 1 Department of Translational Research, Clinical Research Center, Jichi Medical University Hospital, Shimotsuke 329-0498, Japan; 2 School of Medicine, Faculty of Medicine, Gunma University, Maebashi 371-8511, Japan; 3 Jichi Medical University, Shimotsuke 329-0498, Japan; 4 Clinical Pharmacology Center, Jichi Medical University Hospital, Shimotsuke 329-0498, Japan

**Keywords:** aortic dissection, Myh11, trans-omics

## Abstract

Familial thoracic aortic aneurysm and dissection (FTAAD), caused by the pathogenic Myh11 K1256del variant, is characterized by impaired aortic contractility; however, how reduced contractility predisposes the aorta to dissection remains incompletely understood. In this study, we performed a data-driven trans-omic upstream analysis using Genome Enhancer to identify key regulatory mechanisms in aortas from Myh11 K1256del mice under baseline conditions, without exposure to exogenous pathological stimuli. Transcriptome analysis revealed enrichment of genes related to smooth muscle contraction and regulation of myosin light chain phosphatase activity. Upstream computational analysis of regulatory regions identified nuclear factor of activated T cells 1 and lymphoid enhancer-binding factor 1 as major transcription factors, and further highlighted Rho-associated, coiled-coil-containing protein kinase 1 (ROCK1) as a predicted central regulator of the dysregulated transcriptional network. Druggability analysis suggested ROCK1 and the JunB proto-oncogene AP-1 transcription factor subunit as potential therapeutic targets. Furthermore, it predicted 51 candidate therapeutants, including atorvastatin, GSK-269962A, and atovaquone. These findings indicate that even in the absence of overt pathological stimulation, aortic tissue carrying the Myh11 K1256del variant exhibits a transcriptional program centered on ROCK signaling, which may prime the aorta for maladaptive responses to additional stress and may enhance susceptibility to dissection. This computational analysis requires experimental validation, but may provide a hypothesis-generating framework for development of preventive pharmacological interventions against FTAAD.

## 1. Introduction

Aortic dissection is a catastrophic clinical event, in which blood surges into the aortic wall, dissecting the media and the adventitia [1]. At least 20% of non-syndromic thoracic aortic aneurysm and dissection (TAAD) cases exhibit familial aggregation, known as familial thoracic aortic aneurysm and dissection (FTAAD) [2,3,4]. Several key genes involved in FTAAD onset have been identified, including actin alpha 2, smooth muscle (ACTA2) [5], myosin heavy chain 11 (MYH11) [6], myosin light chain kinase (MYLK) [7] and cyclic GMP-dependent protein kinase (PRKG1) [8], which encode functional molecules involved in vascular smooth muscle cell (VSMC) contraction. *MYH11* encodes the smooth muscle-specific myosin heavy chain, and its pathogenic variants generally occur in the C-terminal coiled-coil domain, causing polymerization of thick filaments and VSMC contraction to fail [9]. The frequency of the *MYH11* variant is approximately 1% of all FTAAD patients [6]. We identified a single lysine deletion in Myh11 in two FTAAD families [10].

By establishing an FTAAD mouse model carrying the corresponding lysine deletion (K1256del), we discovered several defects that may contribute to the onset of aortic dissection [11,12,13]. Phenotypically, aortas from mice carrying the K1256del pathogenic variant in both alleles (Myh11^ΔK/ΔK^) exhibited reduced contractile response to vasomotor stimulation [11]. Myh11^ΔK/ΔK^ aortas showed downregulation of integrin subunit alpha 2 (Itga2), which implies impairment of cell adhesion [11]. Investigation combining transcriptomics and metabolomics revealed that downregulation of multiple genes encoding membrane transporters, including calcium channels and ADP-ribose synthesis, which increase cytosolic calcium ion concentration, occurs in Myh11^ΔK/ΔK^ aortas [12]. Proteomic analysis revealed that multiple proteins, transcription of which is regulated by zyxin, were downregulated, and that Myh11 interacted with components of the focal adhesion complex to which zyxin binds [13]. Thus, it was proposed that K1256del-induced Myh11 misfolding disrupts recruitment of zyxin to the focal adhesion complex, impairing zyxin-mediated transcriptional activation, which triggers a cascade of aberrant gene expression in Myh11^ΔK/ΔK^ aortas [13]. Despite these insights into molecular components of FTAAD, it remains uncertain how these pathological events induce specific intracellular pathways that lead to the onset of aortic dissection.

A growing number of studies have employed upstream analysis to identify regulators of pathogenic gene expression patterns in various diseases [14,15,16,17]. Upstream analysis comprises the following steps: (1) Identifying transcription factors (TFs) involved in regulation of differentially expressed genes; (2) reconstructing signal pathways that activate those identified TFs; and (3) identifying master regulators upstream of these pathways [15]. Furthermore, the utility of upstream analysis has been demonstrated in cardiovascular contexts as well. A previous study identified TEA domain transcription factor 1 (TEAD1) as a transcription factor regulated by the heart failure-associated protein, Wnt5a [18]. That study revealed the Wnt5a-TEAD1/YAP axis as a mechanotransduction pathway mediating cardiac responses to mechanical stress, suggesting that TEAD1 is involved in maladaptive remodeling and represents a potential therapeutic target for heart failure [18].

Rho-associated coiled-coil-containing protein kinase 1 (ROCK1) is serine/threonine kinase activated by binding to Ras homolog family member A (RhoA) [19]. ROCK activation induces phosphorylation of several proteins such as myosin phosphatase targeting subunit 1 (MYPT1) and CPI-17, involved in regulation of SMC contraction [20]. Phosphorylation of MYPT1 regulatory subunit MLCP inhibits phosphatase activity of MLCP [20]. When dephosphorylated, active MLCP induces dephosphorylation and relaxation of myosin light chain. The increase in MYPT1 phosphorylation presumably increases myosin contraction [20].

In the present study, we used Genome Enhancer, an automatic pipeline for upstream analysis, to reveal new potential mechanisms for aortic dissection onset Myh11^ΔK/ΔK^ mice. We identified transcription factors and master regulators of differentially expressed genes in aortas of Myh11^ΔK/ΔK^ mice. Genome Enhancer also found master regulators that may represent potential therapeutic targets and proposed drug candidates for FTAAD.

## 2. Results

### 2.1. Functional Classification of the Transcriptome

**Figure 1:**
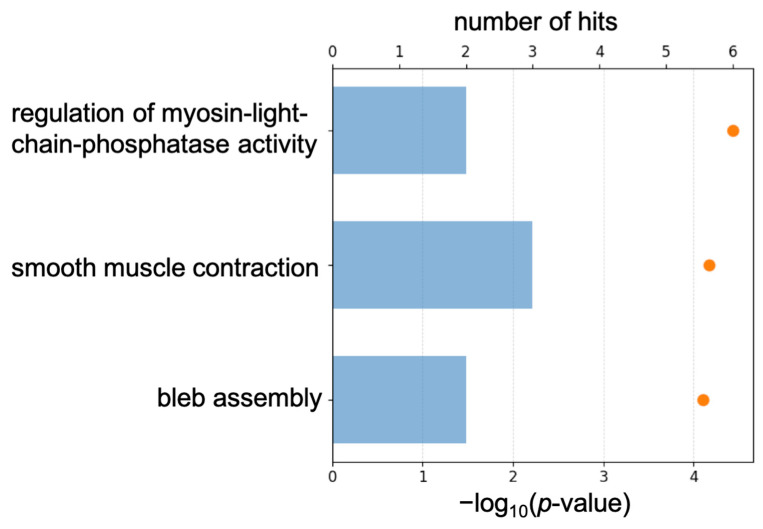
Summary of Gene Ontology analysis, showing three pathways in which upregulated genes were most enriched. Orange dots indicate −log_10_(*p*-value) and blue bars indicate number of hits.

To characterize transcriptomic-level changes, we analyzed transcriptomic data to identify differentially expressed genes and enriched pathways in Myh11^ΔK/ΔK^ aortas. We calculated *p*-values and base two logarithms to the fold change between control and Myh11^ΔK/ΔK^ mice. Transcriptomic analysis identified 94 upregulated genes (logFC > 0.5), including 25 that were significantly upregulated (*p* < 0.01) (Supplementary Table S1). Furthermore, pathway analysis using the Gene Ontology (GO) database showed that 64 pathways were enriched by those significantly upregulated genes (Supplementary Figure S1). Among those pathways, the three most upregulated pathways were *regulation of myosin-light-chain-phosphatase activity*, *smooth muscle contraction*, and *bleb assembly* (Figure 1).

### 2.2. Identification of Master Molecules and Their Transcription Factors

Next, to uncover transcriptional regulatory mechanisms responsible for dysregulated gene expression in Myh11^ΔK/ΔK^ aortas, regulatory regions of those genes were screened for the presence of transcription factor binding site (TFBS) motifs. Composite module analysis identified two distinct TFBS modules that were recurrently clustered within 1000 bp upstream of transcription start sites of significantly up- or down-regulated genes. Module one of upregulated genes included motifs corresponding to lymphocyte function-associated antigen 1 (LFA-1), lymphoid enhancer binding factor 1 (LEF1), (CCCTC-binding factor) CTCF, sterol regulatory element binding transcription factor 2 (SREBF2), and MYC associated zinc finger protein (MAZ). Module two of upregulated genes comprised motifs corresponding to MAZ, LEF1, nuclear factor of activated T cells 1 (NFATC1), and SRY-box transcription factor 10 (SOX10). The resulting composite module model showed strong enrichment in the upregulated gene set compared with the background gene set, with a Wilcoxon *p*-value of 3.68 × 10^−13^ and high classification performance (AUC = 0.95). Transcription factor prioritization based on motif enrichment identified LEF1, SREBF2, and NFATC1 as the top-ranked predicted regulators of upregulated genes (Table 1).

Then, we identified common upstream regulatory factors among identified TF groups. With proteomic data (Supplementary Table S2), we selected differentially expressed proteins involved in signaling pathways and used these proteins as a “context set” in the master regulator identification algorithm [15]. Master regulators were ranked by summing the key node score, the composite module analyst (CMA) score, and log_2_ fold change (logFC) data. These were predicted to exert significant influence on control of intracellular signaling pathways that activate pathological processes of Myh11^ΔK/ΔK^ aortas. Among these master regulators, we chose ROCK1 as the primary master regulator since its total rank and logFC were the highest (Table 2). We also identified transcription factors enhancing the expression of each master molecule. MAZ, CTCF, HNF-1beta, NFATC1, LEF-1, and SREBP-2 were identified as transcription factors for ROCK1. Network visualization of the inferred regulatory cascade (Supplementary Figure S2) facilitated interpretation of upstream analysis results.

### 2.3. Identification of Therapeutic Targets

Next, we evaluated the druggability potential of identified master regulators involved in the pathophysiology of FTAAD. We used the Human PSD^TM^ database of gene–drug assignment and the Prediction of Activity Spectra for Substances (PASS) software, 2020-Standard version (geneXplain GmbH, Wolfenbüttel, Germany) to predict the biological activity of chemical compounds based on (Q)SAR [21,22]. The druggability score represents the number of drugs that are potentially suitable for inhibition or activation of the corresponding target, either according to information extracted from medical literature (from the Human PSD^TM^ database) or according to cheminformatic predictions of compound activity against the examined target (from PASS software). Master regulatory proteins detected as drug targets using Human PSD^TM^ are shown in Table 3, and master regulatory proteins detected based on PASS are shown in Table 4. The most promising therapeutic targets for FTAAD based on druggability scores were ROCK1, phosphorylated myosin phosphatase, and JunB proto-oncogene, AP-1 transcription factor (JunB).

### 2.4. Identification of Drug Candidates

Lastly, we applied algorithms and criteria described in the Materials and Methods to identify drugs that potentially activate or inhibit identified therapeutic targets in the context of certain human diseases. As a result, three drugs approved by the FDA or used in clinical trials for aortopathy were identified (Supplementary Table S3), and 47 repurposed drugs used in clinical trials for other pathologies were identified based on literature curation in the Human PSD^TM^ database (Supplementary Table S4). These drug candidates were ranked based on a composite drug score integrating target activity, disease relevance, and clinical validity. Atorvastatin scored the highest of the drugs approved by the FDA or used in clinical trials for aortopathy (drug score: 52). Among the repurposed drugs identified based on literature curation in the Human PSD^TM^ database, GSK-269962A had the highest drug score of 94. The Human PSD^TM^ database predicted that except for atovaquone, the top five candidates inhibit ROCK1. Atovaquone has the highest drug score among candidates that inhibit JunB activity (Supplementary Table S4). Guanadrel was predicted to be active against MLCP by the PASS software, but the drug score was 0.

## 3. Discussion

This is the first data-driven trans-omic study of FTAAD to identify ROCK1 as a central master regulator that is a potential target for pharmacological intervention. Upstream analysis of the regulatory region revealed that key transcriptional factors such as NFATc1 and LEF-1 may upregulate ROCK1 expression. We prioritized ROCK1 as a computational central master regulator because it ranked highest when considering multiple criteria, including master regulator ranking, druggability, and expression change. While other candidates such as MYPT1 and JunB were also identified, we focused on ROCK1 as a representative regulator to provide a clear and focused mechanistic hypothesis. Finally, druggability analysis identified 51 candidate compounds. Among those, we selected atorvastatin, GSK-269962A, and atovaquone as representative candidate compounds for their high drug score and/or unique targets.

By identifying ROCK1 as a central master regulator, we were able to develop a hypothetical mechanism to explain phenotypes that we observed in our previous study. We previously showed that myosin light chain (MLC) phosphorylation was normal and that contractility was attenuated in Myh11^ΔK/ΔK^ aortas under baseline conditions [11]. Furthermore, Myh11^ΔK/ΔK^ mice did not develop aortic dissection without angiotensin II treatment [11]. In the present study, GO pathway analysis indicated that upregulated genes related to smooth muscle contraction were enriched in Myh11^ΔK/ΔK^ aortas, which may be a compensating mechanism to counteract reduced contractility. The upstream analysis by Genome Enhancer computationally identified ROCK1 as a master regulator, and its gene expression was more active in Myh11^ΔK/ΔK^ aortas. Thus, the normal MLC phosphorylation level previously observed in Myh11^ΔK/ΔK^ suggests that increased *Rock1* transcription did not result in augmented MLC phosphorylation and aortic contraction. This indicates that ROCK1 upregulation alone may be insufficient to restore contractility, likely because compensatory regulatory mechanisms maintain baseline phosphorylation without increasing effective contractile force.

Findings from a previous study comparing conditional and constitutive regulators of calcineurin 1 (*Rcan1*) knockout mice may provide a framework to understand why *Rock1* upregulation in Myh11^ΔK/ΔK^ aortas did not result in increased MLC phosphorylation and recovery of contraction under baseline conditions [23]. That study demonstrated that conditional *Rcan1* knockout in VSMCs increased phosphorylation of MLC in aortas, which was blocked by a ROCK inhibitor, indicating that ROCK-dependent signaling is involved in the recovery of contractility [23]. In contrast, phosphorylation levels of MLC in aortas from constitutive *Rcan1* knockout mice were similar to those in wild-type aortas [23]. This suggests that lifelong upregulation of ROCK1 induced by NFAT, a target of calcineurin, may trigger compensatory adaptations to maintain normal MLC phosphorylation during development or maturation in constitutive *Rcan1* knockout mice [23]. Since the lysine deletion in Myh11^ΔK/ΔK^ is constitutive, *Rock1* upregulation is expected to be lifelong as well. Thus, in future studies, it may be worth investigating whether a similar phosphorylation-buffering mechanism exists under baseline conditions, maintaining normal MLC phosphorylation despite increased *Rock1* expression.

Compensatory upregulation of *Rock1* may be explained by increased mechanical stress resulting from reduced contractility. Computational modeling showed that reduced contractility resulted in significant changes in aortic wall behavior, such as increased transition strain, altered shear properties, and increased rupture and dissection strain in VSMCs [24]. Furthermore, a study with aortas from aged mice revealed that aortas with impaired contractility exhibited lower capacity to counteract deformation induced by pressure loading, thereby increasing mechanical stress by stretching [25]. Among transcriptional factors that we identified as enhancing transcription of *Rock1*, NFATc1 and LEF-1 can be linked to activation by mechanical stress. Calcineurin, which dephosphorylates NFATc1 and induces NFATc1 translocation, requires a rise in cytosolic Ca^2+^ concentration for activation [26]. Aortic VSMCs express stretch-activated calcium channels [27], and intense mechanical stretching in Myh11^ΔK/ΔK^ may cause augmented Ca^2+^ influx through stretch-activated calcium channels. In fact, a previous report demonstrated that mechanical stretching increased calcineurin activity [28]. In the same study, leptin treatment also induced calcineurin activation and NFATc1 translocation [28]. LEF-1 enhances transcription by binding to β-catenin in the nucleus [29]. Phosphorylation of β-catenin is hindered by interaction with E-cadherin [30]. It was experimentally shown that mechanical stretching opens the phosphorylation site, and subsequent phosphorylation by Src kinase released β-catenin to the cytosol [30]. Then, β-catenin translocated to the nucleus [30]. Shear stress induced translocation of β-catenin to the nucleus, where it bound to T-cell-specific factor, a transcription factor related to LEF-1 [31]. Cyclic stretch promoted β-catenin nuclear translocation, but did not upregulate expression of the gene encoding LEF-1 [32]. Thus, increased mechanical stress on VSMCs induced by reduced contractility may promote the expression of ROCK1 by activating the transcription factor activity of NFATc1 and LEF-1.

As discussed above, ROCK1 upregulation did not recover contractility. Thus, mechanical stress may continuously activate *Rock1* transcription by NFATc1 and LEF-1, leading to ROCK1 accumulation. Further, angiotensin II or reactive oxygen species activate RhoA/ROCK signaling [33,34,35,36]. We propose that ROCK1 upregulation may represent a primed or adaptive state, rather than directly restoring contractile function under baseline conditions. In this scenario, ROCK1 activation, rather than its expression level alone, may contribute to pathological responses, particularly upon exposure to additional pathological stimuli such as angiotensin II or oxidative stress that activate RhoA/ROCK signaling. Future studies need to experimentally determine whether additional pathological stimuli, such as angiotensin II or reactive oxygen species, can overly activate ROCK1 and phosphorylate MLC when Myh11^ΔK/ΔK^ mice develop aortic dissection.

ROCK1 activation has been linked to apoptosis and aortic fragility. Excessive ROCK1 activation induces nuclear disruption and bleb assembly on the cell membrane through myosin hypercontractility, forming apoptotic bodies [37]. Moreover, activated ROCK recruits and phosphorylates JNK-interacting protein-3 (JIP-3), which triggers c-Jun N-terminal kinase (JNK) activation via phosphorylation [38]. Phosphorylated JNK phosphorylates γ-H2AX and pro-apoptotic proteins, such as B-cell/CLL lymphoma 2 (Bcl-2) interacting mediator of cell death or Bcl-2 modifying factor, stimulating the apoptotic signaling cascade [38,39]. Activated JNK also phosphorylates c-Jun to form activator protein-1 (AP-1) with c-Fos [39]. Activated AP-1 switches VSMCs to a synthetic phenotype, characterized by decreased contractility and enhanced proliferation capacity [40]. Aortic fragility induced by activated AP-1 depends on the balance between proliferation and apoptosis. Excessive apoptosis decreases the number of VSMCs and mechanical strength in the aortic wall [40,41]. Excessive proliferation leads to accumulation of pathological cells, stiffening, and vascular dysfunction, resulting in the onset of aortic aneurysm and dissection [40,41]. Based on these previous observations, we hypothesized that additional pathologic stimuli may activate accumulated ROCK1, leading to excessive downstream signaling, which causes aortic fragility and dissection.

**Figure 2:**
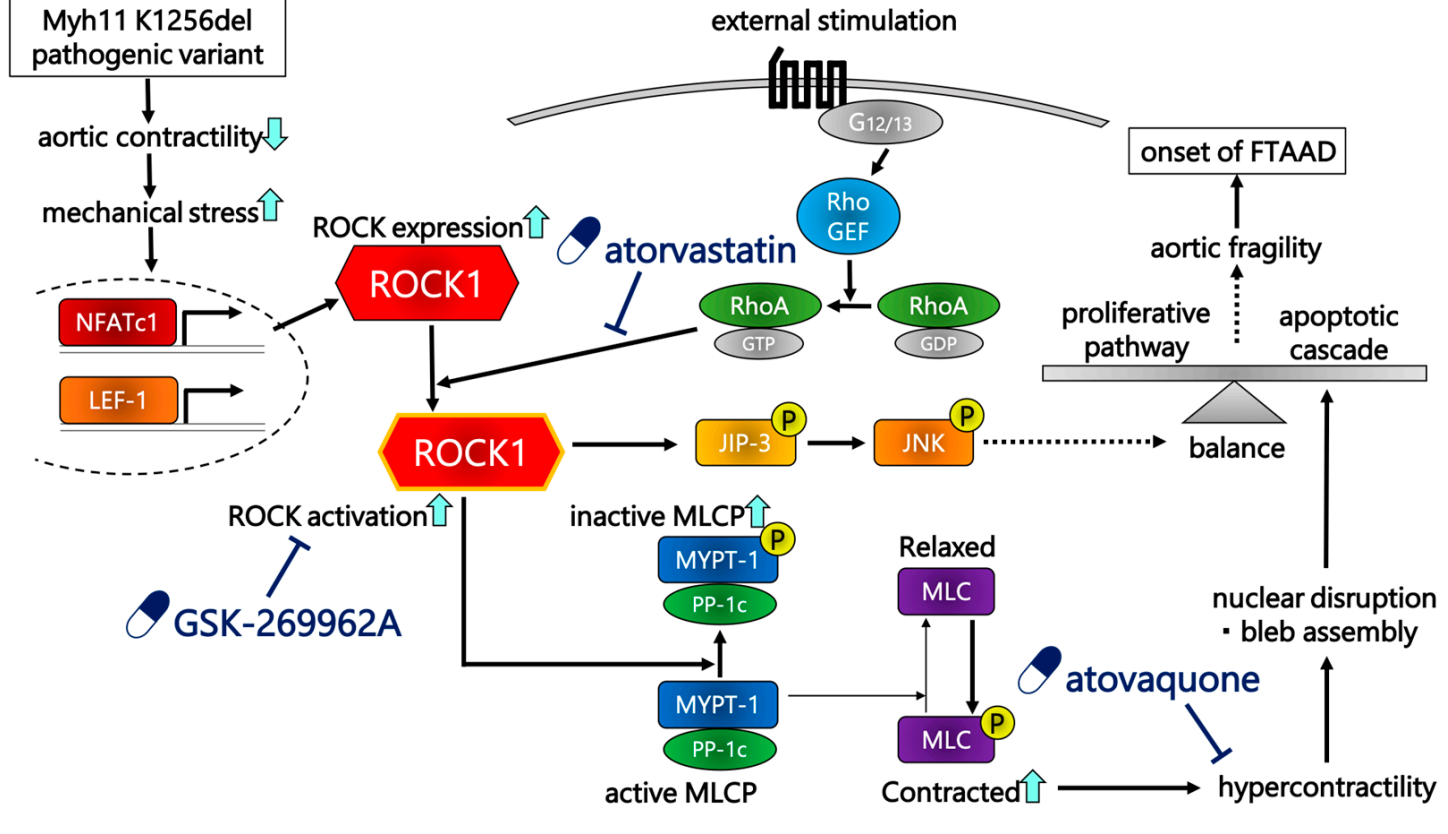
Proposed pathogenic molecular pathways in aortic dissection and targets of drug candidates. NFATc1 = nuclear factor of activated T cell 1; LEF-1 = lymphoid enhancer-binding factor 1; Rho GEF = Rho guanine nucleotide exchange factor; RhoA = Ras homolog family member A; ROCK1 = Rho-associated coiled-coil protein kinase 1; JIP-3 = JNK-interacting protein-3; JNK = c-Jun N-terminal kinase; MYPT-1 = myosin phosphatase targeting subunit 1; PP-1c = protein phosphatase 1 catalytic subunit; MLCP = myosin light chain phosphatase; MLC = myosin light chain. Blue arrows indicate up or downregulation. Flathead arrows indicate inhibition. Dotted **lines** represent conceptual connections rather than molecular interactions.

Three proposed drugs target these ROCK-involved pathways through distinct mechanisms (Figure 2). Atorvastatin is a lipid-lowering drug in the statin class of medications. It competitively inhibits 3-hydroxy-3-methylglutaryl-coenzyme A (HMG-CoA) reductase, a rate-limiting enzyme of the cholesterol biosynthetic pathway [42]. Statins inhibit production of farnesyl pyrophosphate (FPP) and geranylgeranyl pyrophosphate (GGPP), which are produced in cholesterol synthesis pathways and mediate protein prenylation [42]. Prenylation of Rho protein increases ROCK activation through RhoA/ROCK signaling [42]. Thus, statins inhibit ROCK activation in patients with multiple cardiac diseases such as atherosclerosis, congestive heart failure, hypertension, and coronary artery disease [42].

In addition, atorvastatin exerts pleiotropic effects beyond ROCK inhibition. It upregulates expression of tissue transglutaminase (TGM), which contributes to stabilization of the endothelial basement membrane and promotes the integrity of vessel walls [43]. Furthermore, atorvastatin prevents angiotensin II-induced vascular remodeling by altering collagen and elastin, and it exhibits antioxidant properties by downregulating expression of angiotensin II-induced NADPH oxidase subunit [44]. GSK-269962A is a strong ROCK inhibitor with both ROCK1 and ROCK2 affinity [45].

In clinical use, fasudil is now selected as a ROCK inhibitor. Fasudil is a selective isoquinoline sulfonamide ROCK inhibitor, initially approved for treatment of cerebral vasospasm after subarachnoid hemorrhage, and clinical trials have demonstrated promising therapeutic potential for pulmonary arterial hypertension [46]. Furthermore, ROCK inhibitors can be clinically expected to prevent ROCK-induced catastrophic vascular events, such as expression of ROCK1 in aortas of patients after aortic dissection is upregulated, which is considered a predictor reflecting the risk of cardiovascular events [47].

Atovaquone is an antimicrobial drug indicated for prevention and treatment of *Pneumocystis jirovecii* pneumonia and *Plasmodium falciparum* malaria [48]. Recently, atovaquone has been identified as a novel signal transducer and activator of transcription 3 (STAT3) inhibitor that diminishes gp130 expression and decreases availability of key proteins involved in STAT3 activation [49]. Since activated STAT3 promotes the transcription of JunB [50], atovaquone is expected to suppress JunB expression. JunB is a component of activator protein-1 (AP-1) transcription factor and may contribute to smooth muscle cell (SMC) contractility, which changes actin polymerization and myosin light chain phosphorylation [51]. Thus, suppression of JunB expression is expected to inhibit excessive VSMC contraction and aortic fragility. Atovaquone currently lacks established evidence in the treatment of cardiovascular disease. However, our data-driven approach aims to identify potential therapeutic candidates beyond conventional indications, which may reveal previously unrecognized mechanisms. Therefore, atovaquone should be considered a hypothetical candidate requiring further experimental validation, rather than an immediately translatable therapeutic option.

Furthermore, the identified compounds are computationally predicted candidates. Their therapeutic efficacy and relevance to FTAAD require experimental validation in appropriate in vitro and in vivo models before clinical consideration. Together with atorvastatin’s pleiotropic effects and atovaquone’s lack of established evidence relevant to cardiovascular disease, these agents should be interpreted as hypothetical candidates that need further validation, rather than established therapeutic options.

Considering their pharmacological mechanisms of action, these drug candidates are expected to serve as preventive interventions against development of aortic dissection, rather than as treatments subsequent to its onset. Specifically, these agents are thought to mitigate aortic injury responses triggered by external stimuli. Predicted mechanisms of action primarily involve inhibition of ROCK1 overactivation or its downstream pathways, rather than promotion of wound healing or provision of structural support. Accordingly, the anticipated clinical approach involves screening patients with a family history of aortic dissection for *MYH11* pathogenic variants. Upon identification of such variants, one of these candidate agents may be administered prophylactically prior to the onset of aortic dissection. However, to enable this strategy, it is necessary to evaluate therapeutic efficacy to reveal precise mechanisms of action. Importantly, long-term safety and optimal routes of administration must be established, as lifelong treatment would likely be required; thus, non-invasive or long-acting formulations are preferable to daily injections [47].

Although our study used a trans-omic approach to provide novel insights into FTAAD, several limitations remain. Our findings are hypothetical and are based primarily on data-driven computational predictions. Further studies are required to assess ROCK1 activity, downstream signaling, and the effects of pharmacological intervention in the FTAAD.

The temporal relationship between ROCK signaling and structural degeneration cannot be determined from the present study, as our analysis was based on baseline omics data rather than a time-course study. Our proposed model is derived from the functional discrepancy between reduced contractility and preserved MLC phosphorylation, together with transcriptional changes identified in this study. Therefore, ROCK1 upregulation may represent a primed or adaptive state rather than active signaling that drives structural degeneration at baseline. Future studies will be required to investigate temporal dynamics of FTAAD onset and to define the therapeutic window for pharmacological intervention.

We speculated that overactivation of ROCK1 by external stimulation, such as angiotensin II, leads to aortic dissection. Nevertheless, it is possible that adjustment of ROCK1 activity is barely sufficient to compensate for attenuated contractility, rather than pathogenically activating ROCK1.

Although there may be a phosphorylation-buffering mechanism, we would like to emphasize that this concept is hypothetical and based on indirect observation. In the present study, MLCP activity and regulatory components, such as MYPT1, were not directly measured. Instead, MLCP activity was inferred from the lack of increased MLC phosphorylation.

Finally, while we focused on RhoA/ROCK signaling, other significant signaling pathways were not fully considered. The pathophysiology of FTAAD likely arises from complex interactions involving multiple signaling networks. For instance, transforming growth factor β (TGF-β) is involved in multiple pathways including the SMAD, mitogen-activated protein kinase (MAPK), and RhoA/ROCK pathways, promoting production of extracellular matrix in VSMCs, and maintaining the strength of the aortic wall [52]. TGF-β signaling is involved in ROCK activation through engagement of neuroepithelial transforming 1 as a guanine nucleotide exchanger [52]. In specific genetic aortic disease such as Marfan syndrome or Loeys-Dietz syndrome, impairment of TGF-β signaling causes vascular wall fragility and triggers aortic dissection [52]. TGF-β signaling may also participate in the pathophysiology of FTAAD. In fact, our previous proteomic study showed a decrease in TGF-β2 and extracellular matrix. Smooth muscle binding to extracellular matrix was disrupted [13].

In conclusion, we showed that *Rock1* upregulation is likely mediated by activities of transcription factors, especially NFATc1 and LEF-1 in Myh11^ΔK/ΔK^ aortas. Then, we proposed a possible sequence of events leading from reduction in contractility to aortic dissection. Reduced contractility of Myh11^ΔK/ΔK^ aortas may increase mechanical stress, which may activate transcription enhancement of *Rock1* by NFATc1 and LEF-1. We hypothesized that normal MLC phosphorylation levels may be maintained by a phosphorylation-buffering mechanism, possibly overridden by exogenous stimuli such as angiotensin II or reactive oxygen species. In such a case, resulting excessive ROCK1 activation may ultimately induce aortic fragility, preceding dissection. Genome Enhancer also predicted three compounds that inhibit upregulation of pathogenic ROCK1-related signaling. With future studies that assess their efficacy, those compounds may represent a novel medical intervention to prevent patients with Myh11 pathogenic variants from developing aortic dissection.

## 4. Materials and Methods

### 4.1. Data Sets

We retrieved transcriptomic and proteomic data from previous publications [11,13]. Myh11^ΔK/ΔK^ C57BL/6J mice who were 10 to 12 weeks old and their littermate wild-type mice were maintained under a 12 h light/dark schedule [11,13]. Mice were euthanized with a 1100 mg/kg sodium pentobarbital intraperitoneal injection before their aortas were extracted [11,13]. For transcriptomic data, cDNA was prepared from three aortic samples per phenotype (n = three) [11]. Protein was extracted from five aortic samples per phenotype (n = five) [13]. All animal handling procedures in this study complied with the Jichi Medical University Guide for Laboratory Animals and ARRIVE guidelines [53]. The Institutional Animal Care and Concern Committee at Jichi Medical University approved all experimental protocols.

The Limma tool was used to calculate the log_2_ fold change and *p*-value of each gene or protein. Genes with a logFC > 0.5 and a *p*-value < 0.01 were subjected to pathway analysis and upstream analysis. Proteins with a logFC > 0 and a *p*-value > 0.1 were used for upstream analysis.

### 4.2. Analysis of Enriched Transcription Factor Binding Sites and Composite Modules

We analyzed promoters and enhancers of differentially expressed genes for the presence of transcription factor binding sites using DNA binding motifs from the TRANSFAC^®^ library, release 2025.2 (geneXplain GmbH, Wolfenbüttel, Germany) (https://genexplain.com/transfac, accessed on 23 December 2025). These motifs were represented as position weight matrices (PWMs) that describe nucleotide preferences at each position in a binding site.

We defined promoter regions as from −1000 bp to +100 bp relative to the transcription start site. TFBS enrichment was assessed by comparing the frequency of motifs in the differentially expressed gene set (“Yes” set) to a background set of non-differentially expressed genes (“No” set). The Benjamini–Hochberg procedure, with an adjusted *p*-value threshold of <0.01, was used to determine statistical significance. Background expectations were estimated using randomly selected regions of the human genome.

We identified composite regulatory modules using the CMA algorithm [37,38], which detects clusters of TFBSs in sliding windows of 200–300 bp. Modules consisting of up to 10 transcription factors were selected based on their ability to distinguish between Yes and No sets, minimizing the Wilcoxon test *p*-value.

We searched for transcription factor binding sites (TFBSs) that were enriched in promoters and enhancers compared to a background sequence set of promoters of genes that were not differentially regulated under conditions of the experiment. We denoted study and background sets as “Yes” or “No” sets. We considered promoter sequences of 1100 bp (−1000 to +100). The error rate in this part of the pipeline was controlled by estimating the adjusted *p*-value (using the Benjamini–Hochberg procedure) in comparison to the TFBS frequency found in randomly selected regions of the human genome (adj. *p*-value < 0.01). We applied the CMA algorithm to search for composite modules in promoters and enhancers of the Yes and No sets. We searched for composite modules consisting of clusters of 10 TFs in a sliding window of 200–300 bp that separated sequences in the Yes and No sets (minimizing Wilcoxon *p*-value) in a statistically significant manner.

### 4.3. Master Regulator Identification

We used the TRANSPATH^®^ database (BIOBASE), release 2025.2 (geneXplain GmbH, Wolfenbüttel, Germany) (https://genexplain.com/transpath, accessed on 23 December 2025), which provides curated information on signal transduction pathways, for master regulator analysis. A comprehensive signaling network of human cells was constructed based on reactions annotated in TRANSPATH^®^ (see previous studies for main algorithm [15,54]). Upstream regulators were identified by tracing signaling pathways leading to transcription factors detected in the previous analysis. These regulators are considered potential key control nodes capable of influencing large gene expression programs and may represent candidate therapeutic targets. The search was performed with a maximum upstream distance of 12 interaction steps from each transcription factor. To evaluate statistical significance, the analysis was repeated 10,000 times using randomly generated transcription factor sets of identical size. Candidates were selected using a FDR threshold of 0.05 after Z-scores and FDR were computed (see detailed description in [55]).

### 4.4. Analysis of Pharmaceutical Compounds

We identified drug candidates by analyzing interactions between key regulatory network components and known or anticipated medications. The HumanPSD^TM^ database (release 2025.2; geneXplain GmbH, Wolfenbüttel, Germany) (https://genexplain.com/, accessed on 23 December 2025) and PASS provided information on known drugs and their targets.

Compounds linked to at least one molecular target were chosen from the HumanPSD^TM^ database. A composite “drug rank,” which is the total of three factors—the target activity score, the disease activity score, and the clinical validity score—was then used to rank these compounds. The relevance of compound-associated targets to the input gene set is reflected in the target activity score (${T\text{-}score}_{PSD}$), which is computed as follows:

$${T\text{-}score}_{PSD}=-\frac{\left|T\right|}{\left|T\right|+w\left(\left|T_{all}\right|-\left|T\right|\right)}\sum_{t\in T}\log_{10}\left(\frac{rank\left(t\right)}{1+maxRank\left(T\right)}\right),$$

where $T_{all}$ denotes all targets connected to the compound, w is a weighting factor, and T is the set of compound targets that overlap the input list. Each target’s rank is indicated by the term $rank\left(t\right)$, and the maximum rank in the set is denoted by $maxRank\left(T\right)$.

The disease activity score (${D\text{-}score}_{PSD}$) quantifies the clinical relevance of compounds based on associated diseases and their clinical trial phases:

$${D\text{-}score}_{PSD}=\left\{\begin{array}{cc} 0, & \mathrm{if}\;D=\emptyset ;\\ \sum_{d\in D}\sum_{p\in P}phase\left(d,\;p\right), & \mathrm{otherwise}, \end{array}\right.$$

where D is the set of diseases linked to the compound and P represents clinical trial phases. If D is empty, ${D\text{-}score}_{PSD}=0$. The function phase $\left(d,\;p\right)$ returns the phase number if clinical trials exist for disease d in phase p, and zero otherwise.

The clinical validity score corresponds to the highest clinical trial phase (1–4) in which the compound has been evaluated for any indication.

### 4.5. Method for Prediction of Pharmaceutical Compounds

To identify novel candidate compounds with favorable efficacy and safety profiles, we used the PASS program to perform structure–activity relationship (SAR/QSAR)-based analysis. A library of 13,040 chemical compounds with precomputed biological activities, toxicity profiles, and mechanisms of action was examined. All predicted activities were expressed as probabilities of activity (pa).

We selected compounds based on the following criteria:

i. Toxicity probability (defined as pa, probable activity as a toxic substance) below a predefined threshold.
ii. Pharmacological activities relevant to the selected diseases exceeding a defined pa threshold.
iii. At least two anticipated targets (derived from activity mechanisms) with pa values above a specified threshold.

For each compound, the maximum predicted toxicity probability was defined as the “toxicity score”, while the maximum pa value for disease-relevant activities was used as all activities corresponding to selected diseases for a given compound is used as the “disease activity score”. The “Target activity score” (T-score) was calculated as:

$$T\text{-}score\left(s\right)=\frac{\left|T\right|}{\left|T\right|+w\left(\left|T_{all}\right|-\left|T\right|\right)}\sum_{t\in M\left(s\right)}\left(pa\left(m\right)\sum_{g\in G\left(m\right)}IAP\left(g\right)optWeight\left(g\right)\right),$$

where $M\left(s\right)$ denotes the set of predicted activity mechanisms for a given structure s, $G\left(m\right)$ is the set of target genes associated with mechanism m, and $pa\left(m\right)$ is the probability of that mechanism m. $IAP\left(g\right)$ denotes the invariant accuracy of prediction for a given gene g, and $optWeight\left(g\right)$ is an additional weighting factor.

The “Druggability score” (D-score) for a given gene was defined as:

$$D\text{-}score\left(g\right)=IAP\left(g\right)\sum_{s\in S\left(g\right)}\sum_{m\in M\left(s,\;g\right)}pa\left(m\right),$$

where $S\left(g\right)$ is the set of structures associated with a given gene g, and $M\left(s,\;g\right)$ represents the mechanisms that links a given structure to the corresponding gene g.

## Figures and Tables

**Table 1:** Transcription factors for upregulated genes in Myh11^ΔK/ΔK^ aortas predicted by upstream analysis.

Gene Symbol	Gene Description	Regulatory Score ^1^	Yes-No Ratio ^2^
*LEF1*	Lymphoid enhancer binding factor 1	2.45	2.47
*SREBF2*	Sterol regulatory element binding transcription factor 2	2.17	2.66
*NFATC1*	Nuclear factor of activated T cells 1	2.12	6.38
*CTCF*	CCCTC-binding factor	1.95	3.8
*MAZ*	MYC associated zinc finger protein	1.94	2.99
*HNF1B*	HNF1 homeobox B	1.41	2.75
*SOX10*	SRY-box transcription factor 10	0	15.96

^1^ Regulatory score is the measure of involvement of a given TF in the expression control of genes that encode master regulators (presented below) through positive feedback loops. ^2^ Yes-No ratio is the ratio between frequencies of sites in Yes sequences versus those in No sequences. It describes the level of binding site enrichment for the indicated TF in regulatory target regions.

**Table 2:** Top ten master regulators of upregulated genes in Myh11^ΔK/ΔK^.

Master Molecule Name	Total Rank ^1^	LogFC
ROCK1 (h)	10	1.43
Phosphorylated Myosin Phosphatase (h)	10	0.8
Phosphorylated Myosin Phosphatase	11	0.8
MYPT1 (h)	12	0.8
JunB (h)	13	1.79
IGFBP-2 (h)	23	3.27
MSRB3 (h)	28	0.65
PP1-beta (h)	33	0.6
RhoC (h)	37	0.54
PP1-beta (h)	39	0.6

^1^ Total rank is the sum of the ranks of the master molecules sorted by keynode score, CMA score, transcriptomics and proteomics data.

**Table 3:** Prospective therapeutic targets selected from the full list of identified master regulators, filtered by their druggability scores using Human PSD^TM^.

Gene Symbol	Druggability Score	Total Rank ^1^	LogFC
*Rock1*	7	9	1.43
*Junb*	6	11	0.8
*Hspa5*	6	33	0.8
*Tgm2*	18	33	0.8
*Vim*	13	36	1.79
*Atf4*	5	44	0.6

^1^ Total rank is the sum of ranks of master molecules sorted by key node score, CMA score, and log FC.

**Table 4:** Prospective therapeutic targets selected from the full list of master regulators, filtered by their druggability scores using PASS software.

Gene Symbol	Druggability Score	Total Rank ^1^	LogFC
*Ppp1r12a*	0.32	9	1.43
*Ppp1r12b*	0.32	22	0.8
*Ppp1cb*	0.95	28	0.6

^1^ Total rank is the sum of ranks of master molecules sorted by key node score, CMA score, and log FC.

## Data Availability

The raw data supporting the conclusions of this article will be made available by the authors on request.

## Supplementary Materials

The following supporting information can be downloaded at: https://www.mdpi.com/article/10.3390/ijms27073195/s1.
