# Supplementary material for: A Pathogenic ROCK-Signaling Network Involving a Lysine Deletion in Myh11 Renders Carriers Susceptible to Aortic Dissection
Source: Int J Mol Sci. 2026 Mar 31;27(7):3195. doi: 10.3390/ijms27073195 (PMC13072933; doi:10.3390/ijms27073195)
Supplement: Supplementary file 1 [file ijms-27-03195-s001.zip › ijms-4180814-supplementary.pdf]

Supplementary information

for

A Pathogenic ROCK-Signaling Network Involving a Lysine Deletion in Myh11 Renders Carriers Susceptible to Aortic Dissection

Hironori Okuhata [1,2], Shota Tomida[1,2], Tamaki Ishima [1], Ryozo Nagai[3], and Kenichi Aizawa[1,4*]

1 Department of Translational Research, Clinical Research Center, Jichi Medical University Hospital, Shimotsuke, 329-0498, Japan
2 School of Medicine, Faculty of Medicine, Gunma University, Maebashi 371-8511, Japan
3 Jichi Medical University, Shimotsuke, 329-0498, Japan
4 Clinical Pharmacology Center, Jichi Medical University Hospital, Shimotsuke, 329-0498, Japan
* Correspondence: aizawa@jichi.ac.jp

*Address for correspondence:

Kenichi Aizawa, MD, PhD
Jichi Medical University Hospital
3311-1 Yakushiji, Shimotsuke-shi
Tochigi 329-0498, Japan
E-mail: aizawa@jichi.ac.jp

Supplementary Table S1. The table shows upregulated genes in Myh11$^{\Delta K/\Delta K}$ aortas and their $\log_2$ fold change (LogFC) that were input in the upstream analysis by Genome Enhancer.

Genes	LogFC
insulin like growth factor binding protein 2	3.270775
JunB proto-oncogene, AP-1 transcription factor subunit	1.78911
Rho associated coiled-coil containing protein kinase 1	1.427626
sclerostin	1.301228
EF-hand domain family member D1	1.292266
Ras association domain family member 3	1.165421
inhibitor of DNA binding 1	1.147549
cellular communication network factor 3	1.122216
myosin light chain kinase	1.086505
Purkinje cell protein 4 like 1	1.039203
actinin alpha 1	1.020452
nudix hydrolase 4	0.99278
myosin light chain kinase family member 4	0.970907
cysteine and glycine rich protein 2	0.953273
smoothelin	0.903354
BCL2 interacting protein 2	0.891613
calponin 2	0.87703
SH3 domain binding glutamate rich protein like	0.820122
protein phosphatase 1 regulatory subunit 12A	0.800246
potassium channel tetramerization domain containing 10	0.737761
protein phosphatase 1 regulatory subunit 12C	0.722649
LIM zinc finger domain containing 2	0.685578
osteoglycin	0.679294

actinin alpha 4	0.670533
serpin family H member 1	0.588391

Supplementary Table S2. The table shows upregulated proteins in Myh11$^{\Delta K/\Delta K}$ aortas and their $\log_2$ fold change (LogFC) that were input in the upstream analysis by Genome Enhancer.

**Protein**	**LogFC**
LLGL scribble cell polarity complex component 2	32.0266
copine 6	32
myosin binding protein C3	32
toll like receptor 2	32
intraflagellar transport 88	31.99328
regulatory factor X2	31.40146
F-box and WD repeat domain containing 5	29.01457
tripartite motif containing 72	28.01855
RAS p21 protein activator 4	27.84696
cyclin D3	27.23893
pentatricopeptide repeat domain 2	27.10033
vimentin type intermediate filament associated coiled-coil protein	23.71806
TP53 regulated inhibitor of apoptosis 1	17.15724
serine hydrolase like (pseudogene)	10.34108
abhydrolase domain containing 11	0.923826
mitochondrial ribosomal protein L40	0.72116
synaptopodin 2 like	0.700597
deoxyribose-phosphate aldolase	0.698183
mitochondrial ribosomal protein L22	0.676484

dishevelled associated activator of morphogenesis 2	0.59493
DNL-type zinc finger	0.547879

Supplementary Table S3. Prospective drugs approved by the FDA or used in clinical trials for aortopathy identified based on literature curation in HumanPSD™ database

**Name**	**LogFC**
Atrovastatin	32.0266
Acetylsalicylic acid	0.59493
Curcumin	0.547879

Supplementary Table S4. Prospective repurposed drugs used in clinical trials for other pathologies against the identified drug targets based on literature curation in HumanPSD™ database

**Name**	**Target names**	**Drug score**
gsk-269962a	ROCK1	94
belumosudil	ROCK1	92
ripasudil hydrochloride dihydrate	ROCK1	92
Adenine	ROCK1	88
Atovaquone	ATF4,JUNB	87
arundine	ROCK1,VIM	85
pha-793887	JUNB	83
Ibrutinib	JUNB	81
Amphotericin B	TGM2	79
Levamisole	TGM2	77
fimepinostat	JUNB,VIM	75
sotrastaurin	ROCK1	73
Finasteride	TGM2	71

Gemcitabine	TGM2	69
napabucasin	JUNB	67
Isotretinoin	TGM2	65
Antihemophilic Factor (Recombinant)	HSPA5	63
Temoporfin	HSPA5	63
proxyphylline	TGM2	63
5-(1,4-DIAZEPAN-1-SULFONYL)ISOQUINOLINE	ROCK1	58
Deferoxamine	TGM2	56
Dexamethasone	JUNB	54
vactosertib	VIM	52
Decitabine	TGM2	50
Dimethyl sulfoxide	VIM	46
Midazolam	TGM2	44
Camptothecin	TGM2	42
Tolfenamic Acid	HSPA5,ATF4	40
uab-30	TGM2	38
Vitamin A	TGM2	37
Tamoxifen	TGM2	35
Doxorubicin	TGM2	31
Zebularine	HSPA5	29
Progesterone	TGM2	27
marizomib	VIM	25
abt-751	VIM	21
quisinostat	VIM	19
wnt-974	VIM	17
Ethanol	TGM2	15

Ginsenoside Rg1	VIM	13
Niclosamide	VIM	12
Sirolimus	TGM2	10
Triclosan	VIM	8
ginsenoside Rb1	VIM	6
Temozolomide	ATF4	4
delanzomib	ATF4	2
Vitamin E	ATF4	0

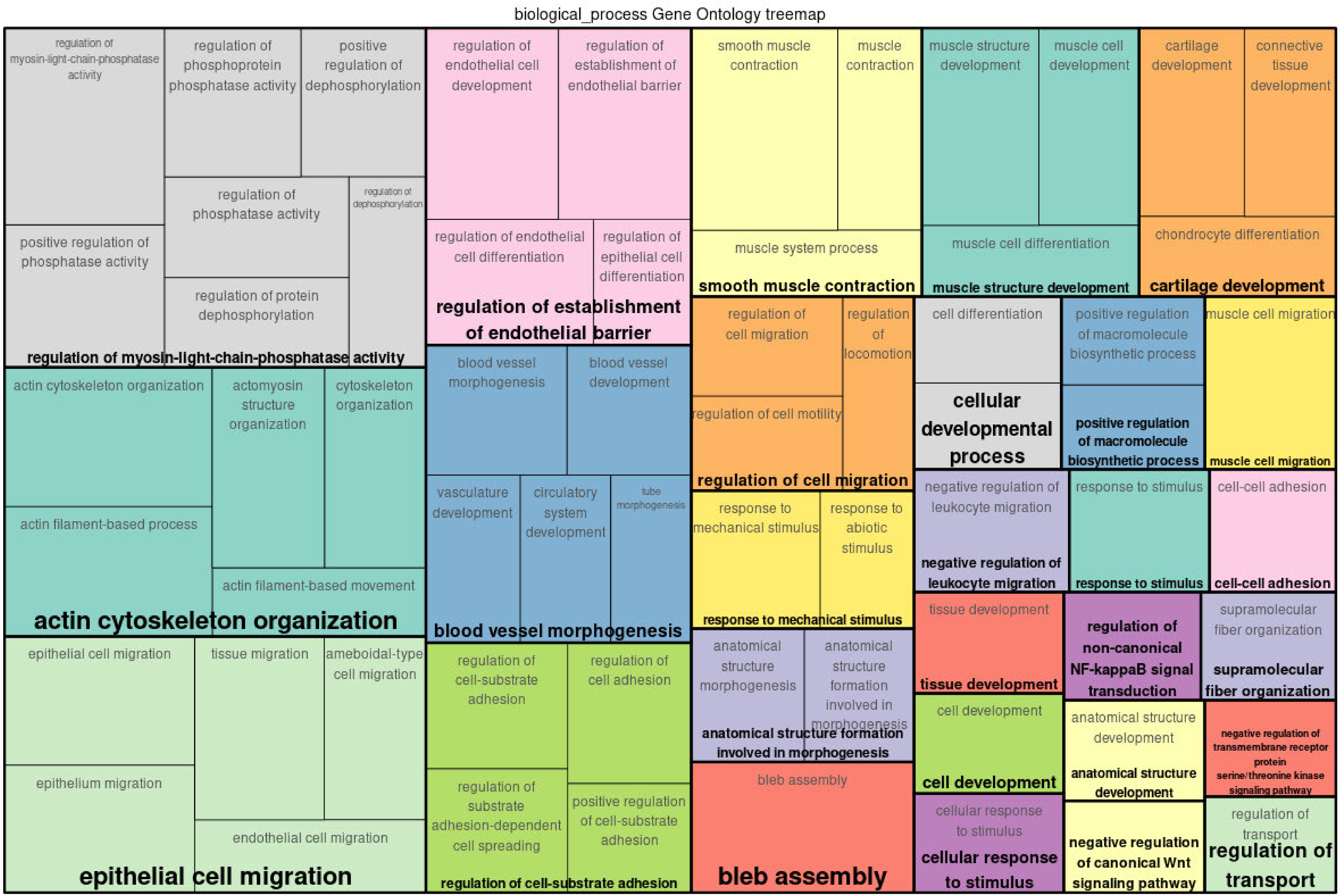
biological_process Gene Ontology treemap
regulation of myosin-light-chain-phosphatase activity
regulation of phosphoprotein phosphatase activity
positive regulation of dephosphorylation
positive regulation of phosphatase activity
regulation of phosphatase activity
regulation of dephosphorylation
regulation of protein dephosphorylation
regulation of myosin-light-chain-phosphatase activity
regulation of endothelial cell development
regulation of establishment of endothelial barrier
regulation of endothelial cell differentiation
regulation of epithelial cell differentiation
regulation of establishment of endothelial barrier
smooth muscle contraction
muscle contraction
muscle system process
smooth muscle contraction
muscle structure development
muscle cell development
muscle cell differentiation
muscle structure development
cartilage development
connective tissue development
chondrocyte differentiation
cartilage development
actin cytoskeleton organization
actomyosin structure organization
cytoskeleton organization
actin filament-based process
actin filament-based movement
actin cytoskeleton organization
blood vessel morphogenesis
blood vessel development
vasculature development
circulatory system development
tube morphogenesis
blood vessel morphogenesis
regulation of cell migration
regulation of locomotion
regulation of cell motility
regulation of cell migration
cell differentiation
cellular developmental process
positive regulation of macromolecule biosynthetic process
positive regulation of macromolecule biosynthetic process
muscle cell migration
muscle cell migration
response to mechanical stimulus
response to abiotic stimulus
response to mechanical stimulus
negative regulation of leukocyte migration
negative regulation of leukocyte migration
response to stimulus
response to stimulus
cell-cell adhesion
cell-cell adhesion
epithelial cell migration
tissue migration
ameboidal-type cell migration
epithelium migration
endothelial cell migration
epithelial cell migration
regulation of cell-substrate adhesion
regulation of cell adhesion
regulation of substrate adhesion-dependent cell spreading
positive regulation of cell-substrate adhesion
regulation of cell-substrate adhesion
anatomical structure morphogenesis
anatomical structure formation involved in morphogenesis
anatomical structure formation involved in morphogenesis
tissue development
tissue development
regulation of non-canonical NF-kappaB signal transduction
supramolecular fiber organization
supramolecular fiber organization
cell development
cell development
anatomical structure development
anatomical structure development
negative regulation of transmembrane receptor protein serine/threonine kinase signaling pathway
bleb assembly
bleb assembly
cellular response to stimulus
cellular response to stimulus
negative regulation of canonical Wnt signaling pathway
regulation of transport
regulation of transport

Supplementary Figure S1. Enriched GO (biological process) of up-regulated genes in Myh11$^{\Delta K/\Delta K}$ aortas vs. wild type aortas. The size of a box is proportional to −log10(P-value).

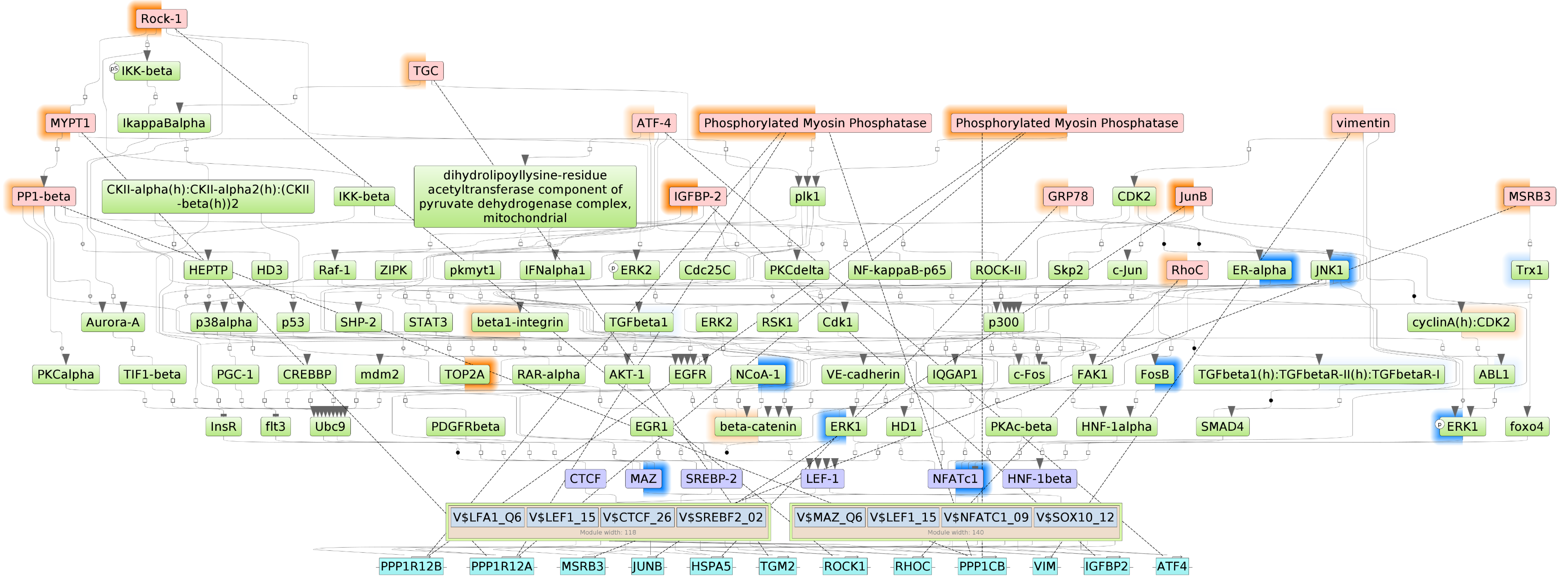
Rock-1
IKK-beta
TGC
MYPT1
IkappaBalpha
ATF-4
Phosphorylated Myosin Phosphatase
Phosphorylated Myosin Phosphatase
vimentin
PP1-beta
CKII-alpha(h):CKII-alpha2(h):(CKII-beta(h))2
IKK-beta
dihydrolipoyllysine-residue acetyltransferase component of pyruvate dehydrogenase complex, mitochondrial
IGFBP-2
plk1
GRP78
CDK2
JunB
MSRB3
HEPTP
HD3
Raf-1
ZIPK
pkmyt1
IFNalpha1
ERK2
Cdc25C
PKCdelta
NF-kappaB-p65
ROCK-II
Skp2
c-Jun
RhoC
ER-alpha
JNK1
Trx1
Aurora-A
p38alpha
p53
SHP-2
STAT3
beta1-integrin
TGFbeta1
ERK2
RSK1
Cdk1
p300
cyclinA(h):CDK2
PKCalpha
TIF1-beta
PGC-1
CREBBP
mdm2
TOP2A
RAR-alpha
AKT-1
EGFR
NCoA-1
VE-cadherin
IQGAP1
c-Fos
FAK1
FosB
TGFbeta1(h):TGFbetaR-II(h):TGFbetaR-I
ABL1
InsR
flt3
Ubc9
PDGFRbeta
EGR1
beta-catenin
ERK1
HD1
PKAc-beta
HNF-1alpha
SMAD4
ERK1
foxo4
CTCF
MAZ
SREBP-2
LEF-1
NFATc1
HNF-1beta
V$LFA1_Q6
V$LEF1_15
V$CTCF_26
V$SREBF2_02
Module width: 118
V$MAZ_Q6
V$LEF1_15
V$NFATC1_09
V$SOX10_12
Module width: 140
PPP1R12B
PPP1R12A
MSRB3
JUNB
HSPA5
TGM2
ROCK1
RHOC
PPP1CB
VIM
IGFBP2
ATF4

Supplementary Figure S2. Diagram summarizing upstream analysis of genes upregulated in Myh11$\Delta^{K/\Delta K}$ aortas compared to Wildtype aortas. Red rectangles = computationally identified master regulators; blue rectangles = transcription factors; green rectangles = intermediate molecules. The orange frames indicate upregulated molecules, whereas the blue frames highlight downregulated molecules. The color frames on the left or right half of molecule names correspond to transcriptomic or proteomic data, respectively.
